# Essential Role of Cdc42 in Ras-Induced Transformation Revealed by Gene Targeting

**DOI:** 10.1371/journal.pone.0037317

**Published:** 2012-06-18

**Authors:** Kristy R. Stengel, Yi Zheng

**Affiliations:** Division of Experimental Hematology and Cancer Biology, Children’s Research Foundation; Department of Cancer and Cell Biology, College of Medicine, University of Cincinnati, Ohio, United States of America; Case Western Reserve University, United States of America

## Abstract

The *ras* proto-oncogene is one of the most frequently mutated genes in human cancer. However, given the prevalence of activating mutations in Ras and its association with aggressive forms of cancer, attempts to therapeutically target aberrant Ras signaling have been largely disappointing. This lack of progress highlights the deficiency in our understanding of cellular pathways required for Ras-mediated tumorigenesis and suggests the importance of identifying new molecular pathways associated with Ras-driven malignancies. Cdc42 is a Ras-related small GTPase that is known to play roles in oncogenic processes such as cell growth, survival, invasion, and migration. A pan-dominant negative mutant overexpression approach to suppress Cdc42 and related pathways has previously shown a requirement for Cdc42 in Ras-induced anchorage-independent cell growth, however the lack of specificity of such approaches make it difficult to determine if effects are directly related to changes in Cdc42 activity or other Rho family members. Therefore, in order to directly and unambiguously address the role of Cdc42 in Ras-mediated transformation, tumor formation and maintenance, we have developed a model of conditional *cdc42* gene in Ras-transformed cells. Loss of Cdc42 drastically alters the cell morphology and inhibits proliferation, cell cycle progression and tumorigenicity of Ras-transformed cells, while non-transformed cells or c-Myc transformed cells are largely unaffected. The loss of Cdc42 in Ras-transformed cells results in reduced Akt signaling, restoration of which could partially rescues the proliferation defects associated with Cdc42 loss. Moreover, disruption of Cdc42 function in established tumors inhibited continued tumor growth. These studies implicate Cdc42 in Ras-driven tumor growth and suggest that targeting Cdc42 is beneficial in Ras-mediated malignancies.

## Introduction

The *ras* proto-oncogene is mutated in approximately 30% of human cancers, including three of the four most deadly cancers in the United States: lung, colon and pancreatic cancers [1], [2]. The Ras protein functions as a central component of multiple mitogenic signaling pathways and regulates a variety of cellular processes including cell growth, differentiation and apoptosis. In response to growth factor signaling, guanine nucleotide exchange factors (GEFs) mediate the cycling of Ras from an inactive, GDP-bound form to an active, GTP-bound form. It is in this GTP-bound form that Ras can interact with a variety of effector molecules, the best characterized of which are the Raf kinase and Phosphatidylinositol 3-kinase (PI3K), to elicit various cellular responses. Ras signaling is terminated by the activity of GTPase activating proteins (GAPs), which accelerate the intrinsic GTPase activity of Ras, returning it to its inactive, GDP-bound form [3]. Cancer-associated mutations in the *ras* gene result in both a diminution of the intrinsic GTPase activity of the Ras protein as well as an insensitivity to GAPs. Therefore, the consequence of Ras mutation is a protein that remains perpetually and aberrantly GTP bound, resulting in constitutively active signaling to downstream effector molecules to promote cell growth and survival associated with cellular transformation.

Given the frequency of Ras activation observed in human cancers, extensive effort has been made to therapeutically target aberrant Ras signaling. However, Ras itself has not been considered a tractable target for small molecule interventions and indirect targeting of Ras activities by farnesyl transferase inhibitors failed to reach the clinic [2], [4]. Major effort has now focused on the targeting of Ras effector pathways such as the Raf-MEK-ERK and PI3K-Akt pathways. Intriguingly, emerging studies examining the molecular mechanisms of Ras-driven transformation have begun to question the impact of the canonical Ras effector pathway, i.e. Raf-MEK-ERK, for Ras-mediated oncogenesis. High throughput RNAi screens designed to identify synthetic lethal interactions in Ras-dependent, K-Ras mutant cancer cells failed to identify genes within the MEK pathway as necessary for cell growth and viability [5], [6], [7]. Cancers expressing oncogenic Ras display varying degrees of sensitivity to MEK inhibition with an overall low level of sensitivity to such inhibitors when compared to cancers harboring BRaf mutations [8]. In addition, a lack of sensitivity to MEK inhibition is consistent with the observation that mutant Ras often does not drive aberrant activation of ERK in cancer cells [9]. Combined, these studies suggest that while signaling to ERK may be crucial for Ras function in normal cells, this pathway may not be the only means of Ras-mediated transformation in cancer cells. Moreover, these data suggest a need to identify additional molecular pathways required for the transforming potential of Ras, as these endeavors may identify other relevant targets for the treatment of cancers driven by oncogenic Ras.

A number of studies have found that the Ras-related small GTPase, Cdc42, becomes activated upon expression of oncogenic Ras [10], [11]. Like Ras, Cdc42 functions as a molecular switch, cycling from an inactive, GDP-bound form to an active, GTP-bound form in response to a variety of extracellular stimuli. Once activated, Cdc42 can regulate cellular processes such as proliferation, actin remodeling, vesicle trafficking and cell polarity [12]. Furthermore, Cdc42 activity has been shown to impinge on Ras-induced signaling pathways including the Raf-MEK-ERK and PI3K-Akt pathways. For instance, while active Cdc42 can bind the p85 subunit of PI3K leading to increased PI3K activity [13], a Cdc42 effector kinase, PAK, may phosphorylate both Raf and MEK to enhance signaling through ERK [14], [15], [16]. Additionally, studies utilizing a dominant-negative Cdc42 mutant suggest that Cdc42 and/or related pathways may be important for Ras-mediated transformation [17].

Here, we present data using a genetic model of Cdc42 loss, which avoids specificity issues commonly associated with the dominant-negative mutant approaches to inhibit Cdc42 function [18], [19], to demonstrate an essential role of Cdc42 in Ras transformation and tumorigenesis. Building on previous biochemical observations, we show that genetic deletion of *cdc42* in Ras-transformed cells results in a significant block in cell proliferation and cell cycle progression, which is not observed in non-transformed cells or cells transformed by the oncoprotein, c-Myc. Further, *cdc42* targeting results in a substantial reduction in Ras-mediated tumorigenesis as well as a reduction in the growth of established tumors, suggesting that Cdc42 activation by oncogenic Ras is crucial for Ras-mediated tumorigenesis and tumor maintenance. Finally, our results implicate Cdc42 as a potentially useful therapeutic target in malignancies harboring activating Ras mutation.

## Results

### Cdc42 is Activated upon Cell Transformation by HRasV12

**Figure 1:**
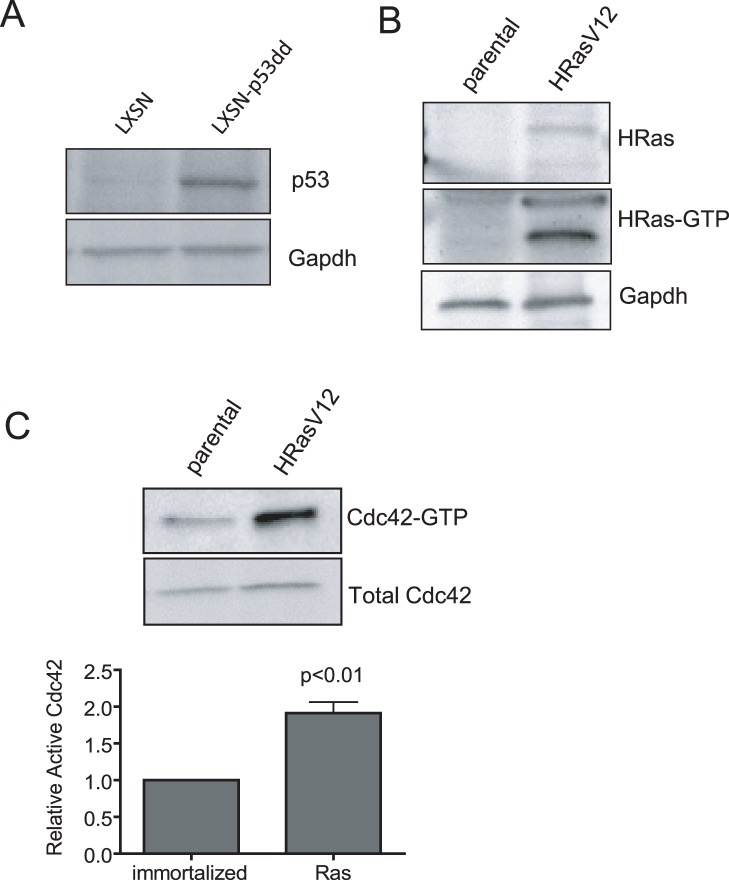
Cdc42 activity is increased upon Ras-mediated transformation. (**a**) Cell immortalization was achieved following retroviral transduction of cdc42^f/f^ cells with a dominant-negative p53 (p53dd). Dominant-negative expression was determined by western blot for p53. (**b**) Immortalized, cdc42^f/f^ cells were transformed through retroviral transduction with HRasV12. Transformed cells exhibit HRas overexpression and increased active, GTP-bound HRas as determined by pull-down with the ras binding domain (RBD) of Raf1 fused to GST. (**c**) HRasV12 transformed cells exhibit a significant increase in Cdc42 activity compared to non-transformed control cells. Pull-down assays were performed with GST-PAK1. The graphed data represents densitometry quantification from three independent experiments.

Studies utilizing a dominant-negative approach to inhibit Cdc42 function in the context of HRasV12-driven cell transformation have suggested that Cdc42 function could be important for the transformation process induced upon aberrant Ras activation [17]. However, given the nonspecific nature of the dominant negative mutant approach, how Cdc42 activity contributes to HRasV12-mediated transformation and whether the targeting of Cdc42 could provide an opportunity for intervention to Ras-mediated transformation have not been rigorously demonstrated. The rationale that Cdc42 inhibition by dominant negative mutants lacks specificity for Cdc42, potentially impinging on the function of other Rho GTPases, is well documented [18], [19]. Here, we have developed a model of inducible *cdc42* gene targeting in HRasV12-transformed fibroblasts as well as non-transformed control cells. Primary mouse embryonic fibroblasts (MEFs) were isolated from *cdc42^loxp^* ^/loxp^ mice, and subsequently immortalized following stable transduction of a dominant negative p53 construct (p53dd) (Figure 1a). Immortalized cells were then transformed through viral transduction of the oncogene, HRasV12. Enhanced activation of the overexpressed oncogene was confirmed by a pull-down assay with GST fused to the Ras binding domain (GST-RBD) of Raf1, and a significant increase in Ras activity in HRasV12-transformed cells relative to p53dd immortalized, non-transformed controls was observed (Figure 1b). Furthermore, consistent with previous reports, cellular transformation by HRasV12 resulted in a significant increase in active Cdc42-GTP level relative to non-transformed control cells (Figure 1c), thus associating active Cdc42 signaling with Ras-driven cell transformation.

### Loss of Cdc42 Results in Dramatic Morphological Changes in Ras-transformed Cells

**Figure 2:**
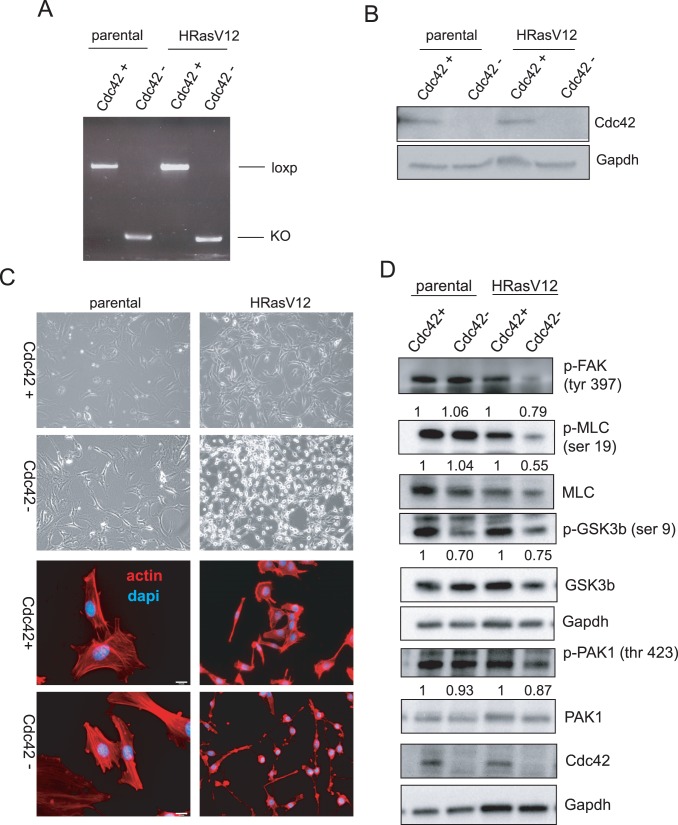
Cdc42 deletion in HRasV12 transformed cells results in dramatic morphological changes and deregulation of Cdc42 effector pathways. (**a**) Recombination of the cdc42 locus following addition of Cre recombinase was monitored by genomic PCR detecting the loxp allele. (**b**) Recombination of the cdc42 locus correlates with loss of Cdc42 protein expression as determined by western blot analysis. (**c**) Cultured HRasV12 cells and non-transformed cells were visualized 4 days following cdc42 deletion by bright field microscopy (upper 4 panels). Cells were fixed and stained with rhodamine-phalloidin and 4,6-diamidino-2-phenylindole (DAPI) to visualize actin structures. (**d**) Cell lysates from Ad-GFP-Cre and control infected cells were immunoblotted for proteins involved in effector signaling downstream of activated Cdc42.

Elevation of Cdc42 activity following overexpression of HRasV12 suggests a role for Cdc42 signaling in cellular transformation by oncogenic Ras. Therefore, to assess the consequence of Cdc42 loss in both parental and Ras-transformed cells, the cells were infected with either adenovirus expressing GFP-Cre to induce recombination of the *cdc42* locus or control adenovirus expressing GFP alone. Cre recombinase expression in both parental and HRasV12-expressing cells resulted in efficient recombination of the *cdc42* locus, as monitored by genomic PCR (Figure 2a). As shown in Figure 2b, efficient *cdc42* gene targeting resulted in the loss of Cdc42 protein expression. Strikingly, while Cdc42 depletion in non-transformed cells led to only modest changes in cell morphology, as demonstrated by brightfield microscopy and actin staining (Figure 2c), the loss of Cdc42 in Ras-transformed cells resulted in a drastic morphological change characterized by rounding of the cell body. However, in spite of these dramatic changes in cell morphology, Cdc42-deficient Ras-transformed cells remained viable as demonstrated by a lack of TUNEL positive cells (Figure S1a). A slight increase in cleaved Caspase 3 levels was observed (Figure S1b) when both adherent and non-adherent cells were harvested for western blot analysis, suggesting that subtle changes in cell viability observed upon Cdc42 loss may be attributed to the reduction in cell adhesion.

### Disruption of Effector Signaling upon Cdc42 Loss

Consistent with the morphological changes observed upon loss of Cdc42 in Ras-transformed cells, reductions in both p-FAK and p-MLC levels, two proteins whose activation is critical for focal adhesion formation and maturation, were observed [20]. In non-transformed cells in which no cell rounding was observed, however, the phosphorylation status of these proteins remained unchanged (Figure 2D). Additionally, oncogene transformed cells are likely addicted to certain signaling pathways, therefore changes in FAK and MLC activities specifically observed in Ras-transformed cells may indicate that Ras-transformed cells are dependent on Cdc42 signaling to activate FAK and MLC, while non-transformed cells may be able to utilize alternative pathways to modulate the activities of these proteins. The Cdc42 effector protein, Par6, binds to Par3/aPKCs to form a complex associated with tight junction formation at cell-cell contacts [21] and cell polarization [22]. Additionally, this complex has been shown to control polarity in migrating cells through the modulation of GSK3β activity following phosphorylation at an inhibitory site, serine 9 [23]. Both non-transformed and Ras-transformed cells exhibited a reduction in phosphorlyation of GSK3β at this site, suggesting disruption of the Par6/Par3/aPKC complex upon Cdc42 loss and regardless of Ras activation status. Further, activation of another established Cdc42 effector protein, PAK1, showed a Ras-specific reduction upon Cdc42 loss. Therefore, multiple pathways downstream of Cdc42 are affected upon Cdc42 deletion in Ras transformed cells.

### Cdc42 Loss Inhibits Cell Growth and Transformation of HRasV12-expressing Cells

**Figure 3:**
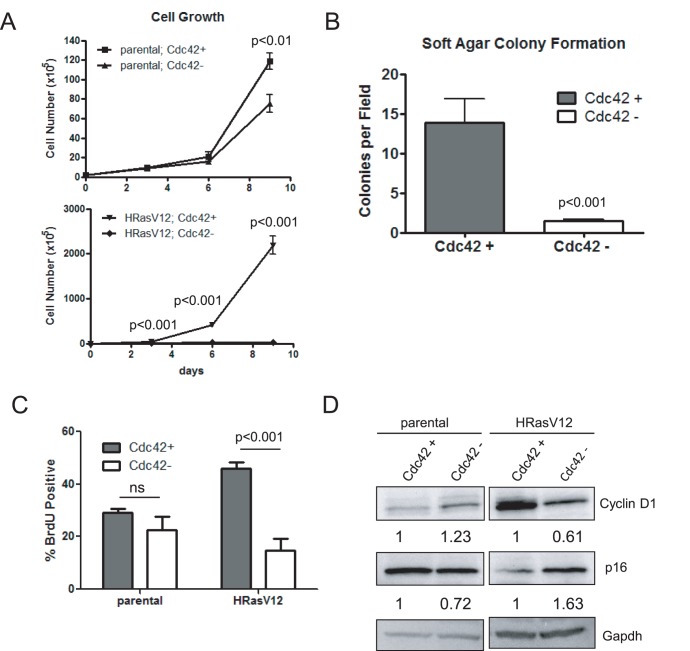
Loss of Cdc42 in HRasV12 transformed cells results in reduced cell proliferation and anchorage-independent growth. (**a**) Equal cell numbers were plated following cdc42 deletion and cells were counted by trypan blue exclusion every three days for 9 days. Curves represent at least two independent experiments performed in triplicate. (**b**) Cdc42-proficient and –deficient HRasV12 cells were grown in a 0.3% agarose solution. Experiments were performed in triplicate and 9 independent fields were counted for colonies. (**c**) Exponentially growing cells were pulsed with 5-bromodeoxyuridine (BrdU) for 1 hr. prior to harvest. Cells were stained with anti-BrdU and propidium iodide (PI) and analyzed by flow cytometry. Graphs represent at least three independent experiments. (**d**) Cell lysates were immunoblotted for the cell cycle regulators, Cyclin D1 and p16^ink4a^. Gapdh served as a loading control.

The Rho family of small GTPases has been shown to be required for G1/S transition [24]. However, previous studies utilizing dominant negative Cdc42 mutants did not observe a growth defect upon mutant expression in Ras-transformed cells [17], and effects of Cdc42 on cell cycle progression appears to be cell type specific as gene targeting of Cdc42 in hematopoietic stem cells or neural progenitors resulted in an increased S-phase transition [25], [26]. Therefore, growth assays were carried out using our inducible *cdc42* gene targeting model to address these apparent discrepancies. While non-transformed cells showed a modest reduction in cell growth over time following Cdc42 deletion (Figure 3a, upper panel), HRasV12-expressing cells exhibited a dramatic and significant reduction in cell growth following Cdc42 loss (Figure 3a, lower panel). To address the ability of Cdc42 loss to inhibit Ras-mediated transformation, soft agar colony formation assays were carried out to assess anchorage-independent growth potential. *Cdc42* deletion resulted in a significant reduction in colony formation (Figure 3b), suggesting that Cdc42 function is crucial for Ras-mediated anchorage-independent cell growth as well as growth in two dimensions. In contrast, Cdc42 overexpression in Ras-transformed cells did not further enhance cell growth, but did lead to enhanced soft agar colony formation with colonies forming more rapidly and growing to a larger size (Figure S2).

In an attempt to address the cellular mechanism of growth reduction upon Cdc42 loss, BrdU assays were carried out to investigate the consequence of Cdc42 loss on cell proliferation. While no significant reduction in BrdU incorporation was observed in parental cells, H-RasV12 expressing cells exhibited a significant reduction in BrdU incorporation following Cdc42 deletion (Figure 3c). Furthermore, Ras-expressing cells showed a reduction in Cyclin D1 and increase in p16^ink4a^ protein levels. Thus, *cdc42* deletion causes a G1 cell cycle arrest in Ras transformed cells (Figure 3d).

### Deletion of Cdc42 Inhibits HRasV12-driven Tumorigenesis

**Figure 4:**
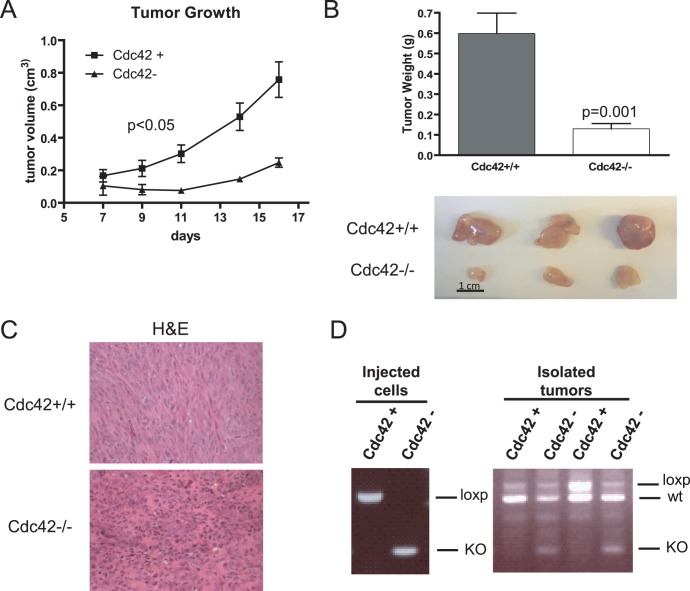
Cdc42-deficiency impairs HRasV12-driven tumorigenesis. (**a**) Cdc42 proficient and deficient cells in a 1∶1 PBS:matrigel mixture were subcutaneously injected into the flanks of immunocompromised nude mice. Developing tumors were measured at indicated time points with calipers to determine tumor volume. n = 6. (**b**) 16 days post-injection mice were sacrificed and tumors excised and weighed (upper panel). A representative image of tumors from three mice are shown (lower panel). (**c**) Tumors were fixed, sectioned and stained with hematoxylin and eosin. (**d**) Left panel - The recombination status of the *cdc42* locus was determined for cells prior to injection into nude mice by genomic PCR. Right panel - DNA was isolated from resulting subcutaneous tumors and the recombination status of *cdc42* determined by PCR. Tumors from two representative mice were shown. The upper band corresponds to the non-recombined loxp/loxp allele from injected cells, the middle band corresponds to the wild-type *cdc42* allele derived from stromal cells derived from the recipient mouse, and the lower band corresponds to the recombined *cdc42* allele.

Given the drastic effects of *cdc42* deletion on cell proliferation and anchorage-independent growth, it seems likely that Cdc42 loss would impair the ability of HRasV12 to drive tumor formation. Therefore, following Cdc42 deletion, cells were injected subcutaneously into the flanks of immunocompromised nude mice. Cdc42-deficiency resulted in a significant reduction in xenograft tumor growth (Figure 4a). Tumors were isolated at the completion of the study and tumors arising from Cdc42-proficient cells grew significantly larger than Cdc42-deficient tumors and exhibited general differences in tumor architecture (Figure 4b & 4c). Interestingly, while genotyping on cells prior to injection into mice seemed to reveal an efficient recombination of the *cdc42* locus, PCR performed on DNA isolated from tumors revealed a resurgence of the non-recombined locus in tumors arising from Cdc42-deficient cells (Figure 4d). This suggests that residual Cdc42-proficient cells remained following Cre-mediated *cdc42* deletion and that those cells were selected for upon injection into mice. It is possible that Cdc42-null cells might more easily dissociate from the tumor or be more susceptible to clearance by the immune system; these results indicate that loss of Cdc42 in oncogenic Ras transformed cells causes a growth disadvantage *in vivo* as well as *in vitro.*

### Cdc42 Status does not Affect the Growth or Transformation of c-Myc-transformed Cells

**Figure 5:**
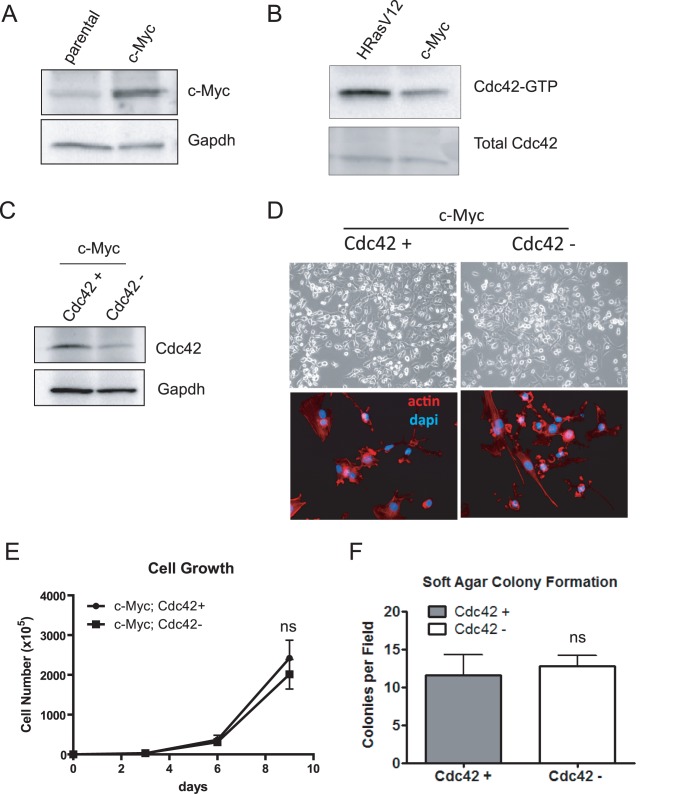
Cdc42 loss does not impair transformation by the oncoprotein, c-Myc. (**a**) p53-immortalized cells were infected with a retrovirus expressing the oncoprotein, c-Myc. c-Myc overexpression was verified by western blot analysis. (**b**) Pull-down assays were performed with GST-PAK1 to determine the relative abundance of activated Cdc42 in HRasV12 vs. c-Myc transformed cells. (**c**) Lysates from c-Myc transformed cells infected with Ad-GFP or Ad-GFP-Cre were immunoblotted for Cdc42. (**d**) Cultured c-Myc transformed cells were visualized 4 days following *cdc42* deletion by bright field microscopy (upper 2 panels). Cells were fixed and stained with rhodamine-phalloidin and DAPI to visualize actin structures (lower 2 panels). (**e**) An equal number of c-Myc transformed Cdc42 proficient and deficient cells were seeded. Cells were counted every three days by trypan blue exclusion. Curves represent at least two independent experiments performed in triplicate. (**f**) Cdc42-proficient and –deficient c-Myc cells were grown in a 0.3% agarose solution. Experiments were performed in triplicate and 9 independent fields were counted for colonies. ns = not significant.

While Ras-transformed cells are exquisitely sensitive to Cdc42 loss when compared to non-transformed control cells, whether Cdc42 is also important for transformation mediated by oncogenes of distinct pathways has not been addressed. c-Myc is another potent and widely studied oncogene which, like Ras, has been shown to cooperate with p53 loss to promote cellular transformation [27]. Cells immortalized by dominant negative p53 were transformed through overexpression of c-Myc (Figure 5a). Comparison of Cdc42 activation status between HRasV12 and c-Myc transformation revealed a reduction in the levels of GTP-bound Cdc42 in c-Myc transformed cells compared to HRasV12-expressing cells (Figure 5b). Furthermore, upon Cre-mediated *cdc42* depletion, c-Myc transformed cells showed no difference in cell morphology (Figure 5d), cell growth (Figure 5e) or anchorage-independent growth (Figure 5f). While these data are not sufficient to conclude that the deleterious effects of Cdc42 loss are exclusive to Ras-transformed cells, they suggest that Cdc42 depletion is not universally detrimental to transformed cells per se, as c-Myc transformation is not dependent on Cdc42 function. Thus, the requirement for sustained signaling through Cdc42 is dependent on oncogenic context. Furthermore, it seems that relatively elevated level of active Cdc42 may be predictive of a cells sensitivity to Cdc42 loss, as the effect of Cdc42 deletion on non-transformed and c-Myc transformed cells is minimal compared to HRasV12-transformed cells which exhibit higher levels of GTP-bound Cdc42 (see Figure 1c and 5b). Consistently, competitive proliferation assays in which a 1∶1 mix of Cdc42 proficient and deficient cells were seeded together and passaged over time, revealed a significant growth advantage of Cdc42 positive over Cdc42 negative HRasV12 cells, while Cdc42 proficiency did not provide a growth advantage for non-transformed or c-Myc transformed cells (Figure S3).

### Growth Defects Associated with Cdc42 Loss are Partially Rescued by Active Akt

**Figure 6:**
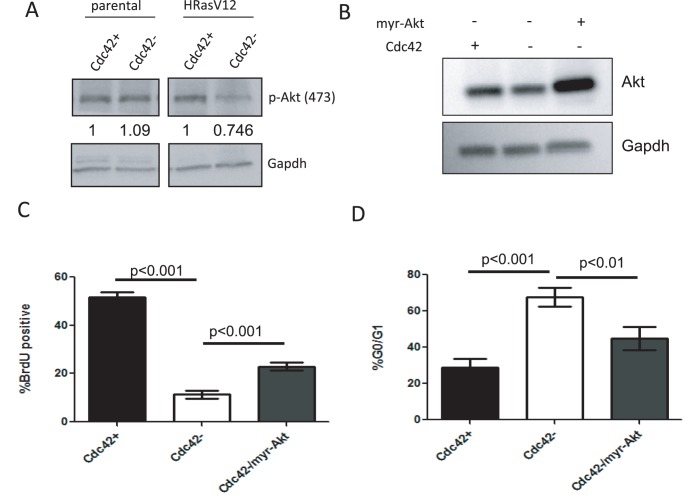
Growth defects observed in HRasV12-transformed cells following Cdc42 loss are partially rescued by activation of Akt signaling. (**a**) Cell lysates from non-transformed and HRasV12-transformed cells were immunoblotted for phospho-Akt. Gapdh serves as a loading control. (**b**) Cells were infected with Ad-GFP, Ad-GFP-Cre or Ad-GFP-Cre + Ad-myr-Akt. Cell lysates were obtained and blotted for Akt expression. (**c**) Exponentially growing cells were pulsed with BrdU for 1 hour prior to harvest. Cells were stained with anti-BrdU and PI and analyzed by flow cytometry. (**d**) PI staining was used to determine the percentage of cells with 2N DNA content. Graphs represent at least three independent experiments.

Since it appears that the loss of Cdc42 is selectively detrimental to HRasV12-transformed cells, the effect of Cdc42 loss on known Ras-effector pathways was examined. We had previously observed that the deletion of Cdc42 in HRasV12-expressing cells results in reduced p-PAK1 levels (Figure 2d), a protein known to regulate activation and signaling through Raf-MEK-ERK [14], [15]. Therefore, we first examined the ability of Cdc42 loss to impinge upon this pathway. Surprisingly, Cdc42 loss did not significantly alter p-MEK or p-ERK levels (Figure S4); however, reproducible reductions in p-Akt levels were observed (Figure 6a and S4), suggesting that Cdc42 deficiency alters signaling through another Ras effector pathway, PI3K-Akt. To assess the significance of reduced Akt activation on the Cdc42 deficient phenotype, a myristoylated, constitutively active Akt construct was transiently expressed in Cdc42-deficient HRasV12 cells (Figure 6b). Cells were subjected to BrdU pulse followed by BrdU and propidium iodide staining. The expression of active Akt resulted in a significant, but partial rescue of the cell proliferation defect observed in Cdc42-deficient cells (Figure 6c). Consistently, myr-Akt was able to partially rescue the G1 growth arrest observed upon Cdc42 loss (Figure 6d). Therefore, while a reduction in Akt signaling contributes to growth defects observed upon Cdc42 loss, there are likely additional mechanisms at play. Alternatively, myr-Akt expression may not result in reconstitution of all Akt functions. Specifically, expression of myr-Akt results in constitutive Akt localization at the cell surface where it is activated; however, Akt has also been shown to have nuclear functions [28] which would not be restored by such a mutant.

### Cdc42 Deletion Inhibits HRasV12-driven Tumor Growth

**Figure 7:**
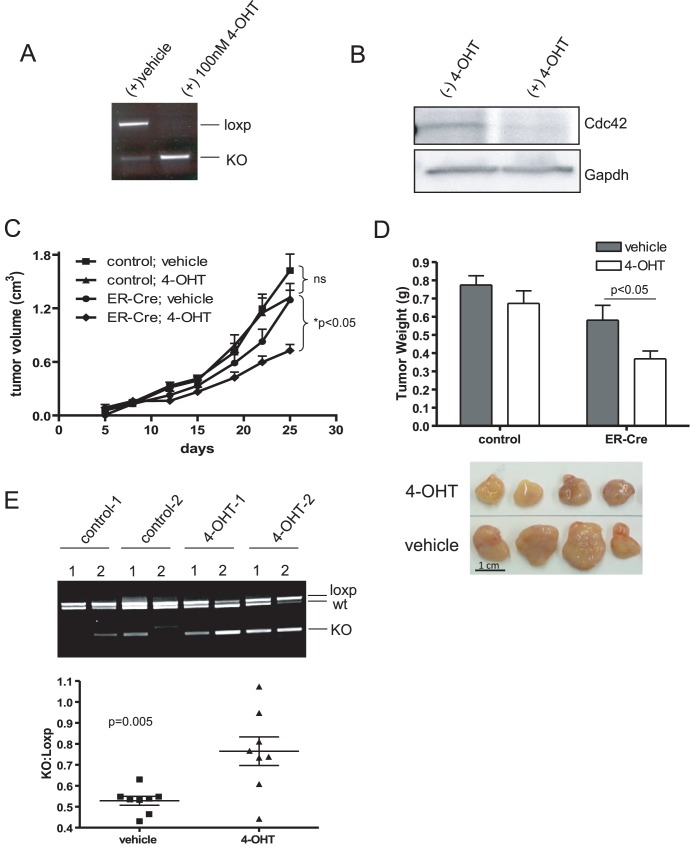
Cdc42 deletion in HRasV12-driven tumors inhibits tumor growth. (**a**) HRasV12; Cdc42^f/f^ cells were transduced with retroviral ER-Cre and selected with puromycin. Antibiotic resistant cells were pooled and treated in culture with 4-OH Tamoxifen (4-OHT) or vehicle. Recombination at the *cdc42* locus was determined by PCR. (**b**) Cell lysates were isolated from cells cultured in the presence and absence of 4-OHT. Western blotting was performed to determine Cdc42 protein levels following Tamoxifen-induced recombination. (**c**) HRasV12; ER-Cre; Cdc42^f/f^ and HRasV12; Cdc42^f/f^ cells were injected into the flanks of immunocompromised nude mice. Five days post-injection, palpable tumors had formed and mice were administered 100 µl of 10 mg/ml 4-OHT in sunflower seed oil or vehicle control once a day, five days a week for 3 weeks. Tumor volume was monitored by caliper measurements. n = 8. (**d**) At 25 days post-injection, mice were sacrificed and tumors excised and weighed. A representative image of 4 HRasV12; ER-Cre; Cdc42f/f tumors treated with either tamoxifen or vehicle control is shown. (**e**) Genotyping was performed on DNA isolated from HRasV12; ER-Cre; Cdc42^f/f^ tumors treated with either vehicle or 4-OHT to determine the status of the *cdc42* locus. A representative image of genotyping results from two mice per condition (two tumors per mouse) is shown. Densitometry was performed using imageJ software and the intensity of the recombined *cdc42* allele relative to the non-recombined *cdc42* allele is plotted.

While constitutive deletion of *cdc42* inhibits tumor formation and subsequent growth, to address the feasibility of targeting Cdc42 for therapeutic intervention in tumors driven by HRasV12 mutation, *cdc42* was inducibly deleted in established tumors. HRasV12; Cdc42f/f cells were stably transduced with Cre-ER^T^, such that *cdc42* could be conditionally deleted upon tamoxifen administration (Figure 7a) resulting in a near complete reduction of Cdc42 protein (Figure 7b). It is worth noting that the ER-Cre allele is somewhat leaky, allowing for modest amounts of recombination even in the absence of tamoxifen (Fig. 7A, 7E). HrasV12; Cdc42f/f cells +/− Cre-ER^T^ were injected into the flanks of immunocompromised nude mice. Five days post-injection, palpable tumors had formed, at which time, mice were administered either 4-OH tamoxifen or vehicle control once daily, five days a week for the duration of the study. While tamoxifen treatment alone did not inhibit the growth of tumors lacking the Cre-ER^T^ construct compared to vehicle controls, Cre-ER^T^ positive tumor growth was significantly inhibited by tamoxifen administration (Figure 7c). Furthermore, at the end of the assay, tamoxifen-treated, Cre-ER^T^ positive tumors were significantly smaller compared to controls (Figure 7d). Interestingly, while tamoxifen treatment significantly enriched *cdc42* recombination in treated tumors, it was not able to induce complete deletion of the gene (Figure 7e), further supporting the notion that Cdc42-deficiency is counter-selected and functional Cdc42 signaling is critical for Ras-driven tumorigenesis.

## Discussion

Activated Cdc42 mutants are capable of transforming immortalized fibroblasts, suggesting a role for Cdc42 in oncogenesis [29]. While mutations in Cdc42 have not been reported in human cancer, upregulation of Cdc42 protein expression or activity has been reported in a variety of tumor types and in some instances have been correlated with poor prognosis [30], [31], [32], [33]. However, how Cdc42 activity contributes to the transformed phenotype and cooperates with other oncogenic events has not been rigorously explored. Furthermore, additional studies are needed to assess the consequences and feasibility of targeting Cdc42 signaling in distinct tumorigenic and tumor maintenance contexts. Here, we report that Cdc42 is critical for oncogenic Ras-driven cell transformation and tumor growth. Consistent with studies using dominant-negative Cdc42 mutants [17], we found that the loss of Cdc42 dramatically inhibits the ability of HRasV12 to promote cellular transformation, as determined by soft agar colony formation. However, in contrast to dominant-negative studies, we reveal that Cdc42 loss results in a significant reduction in cell growth, attributable to a G1 phase cell cycle arrest. Interestingly, this observation is consistent with a previous report that suggested a requirement Cdc42 for progression into S phase by dominant negative mutant expression [24]. The current work is significant because it unambiguously demonstrates the essential role of Cdc42 activity in Ras-mediated transformation and tumorigenesis by a genetic means, as previous approaches utilizing overexpression of a mutant Cdc42 may very well introduce non-specific artifacts due to cross-reactivity of dominant negative Cdc42 mutant with multiple upstream Rho GEFs, thus affectingother Rho GTPases and related signaling networks. In addition to studies carried out in rodent cells, transformation of human fibroblasts with HRasV12 is also inhibited upon dominant negative Cdc42 expression. Interestingly, expression of angiogenic factors was downregulated upon Cdc42 inhibition in this model [34]. However, while this effect may account for differences in tumorigenesis *in vivo*, it does not explain *in vitro* changes in cell growth observed upon Cdc42 inhibition.

Interestingly, the level of Cdc42 activity was found to be significantly enhanced upon HRasV12 expression compared to non-transformed or c-Myc transformed cells, suggesting that Cdc42 can be specifically activated by oncogenic Ras. Consistent with the idea that oncogenic Ras signaling renders cells “addicted” to Cdc42 activation, HRasV12-transformed cells are exceedingly sensitive to Cdc42 deletion, while non-transformed cells or cells transformed by c-Myc overexpression are minimally affected by Cdc42 loss. This further suggests that Cdc42 dependence is restricted to certain oncogenic contexts, such as those induced upon oncogenic H-Ras activation.

In addition to changes in cell growth, we observed a dramatic change in the morphology of HRasV12-transformed cells upon Cdc42 loss, which correlated with a reduction in the activation of the focal adhesion protein, FAK. One recent study by Zheng et. al, suggested that phosphorylation of FAK downstream of Ras is inhibited to promote Ras-induced cell migration and that the reduction in phosphorylated FAK requires the activity of Cdc42 [11]. Interestingly, we also see a decrease in p-FAK levels following Ras transformation (Fig. 2d); however p-FAK levels are largely absent following Cdc42 deletion. This seeming discrepancy could possibly reflect a dosing effect, as the previous study utilizes an siRNA-mediated silencing of Cdc42, which often leaves residual protein, while our targeted gene deletion approach eliminates Cdc42 signaling to a point which is not conducive to continued signaling to FAK. Alternatively, this could represent a cell-type specific effect. However, it is worth noting that, while the mechanism proposed by Zheng *et al.* for Cdc42-induced FAK inhibition involved activation of ERK signaling downstream of active Cdc42, we did not observe any appreciable disruption of either MEK or ERK activation upon Cdc42 loss (Figure S4). This observation is consistent with previous reports that demonstrated activation of JNK signaling, but not ERK signaling, upon Cdc42 activation [24], [35].

While changes in the phosphorylation status of MEK and ERK were not observed in HRasV12-expressing cells following Cdc42 loss, reduction in the levels of p-Akt was evident, suggesting that Cdc42 depletion may compromise the activation of PI3K signaling downstream of activated Ras. This is consistent with reports that GTP-bound Cdc42 can interact with the p85 subunit of PI3K to modulate PI3K activity [13]. Recently, Ras was reported to associate with Cdc42 at endomembranes, while Ras molecules restricted to the plasma membrane were able to maintain signaling to Raf [10]. It is tempting to hypothesize that Cdc42 may modulate Ras-mediated transformation by altering signaling to PI3K at endomembranes. However, complementing Cdc42 deficient cells with a constitutively active Akt was only able to partially restore proliferation of Cdc42 deficient cells, indicating that additional mechanism may exist by which disruption of Cdc42 signaling hinders Ras-induced cell growth. Aside from discrete signaling activities of Ras localized at various cellular compartments, restriction of Ras localization also appears to impact the ability of Ras to promote transformation [10], [36], [37]. To this end, whether Cdc42 loss impacts subcellular localization of oncogenic Ras has not been explored. Interestingly, Cdc42 has been previously shown to modulate EGFR-induced transformation by regulating its localization and turnover [38], [39], establishing a precedence for Cdc42-mediated changes in transformation following modulation of oncogene localization and stability.

Understanding the signaling events necessary for the transforming activities of Ras is crucial for the development of novel Ras-targeted therapies. Emerging studies have begun to explore the therapeutic values of various targeting strategies outside the canonical Ras effector pathway, Raf-MEK-ERK, and emphasize the significance of identifying additional pathways either directly involved in Ras signaling or functioning as key modifiers that are required for oncogenic Ras-driven tumorigenesis [5], [8], [9]. Whether and how Cdc42 and its related pathways contribute to Ras-mediated transformation and tumor maintenance in primary human pathologies will be an issue of further investigation. In particular, given the well appreciated cell-type specific signaling function of Cdc42 [40], it can be anticipated that the proof of principle shown here may apply to specific tissue/cell types where Ras-induced transformation may be intimately dependent on Cdc42. Additionally, while the described studies specifically identify a requirement for Cdc42 in HRasV12-driven transformation, how Cdc42 might affect transformation resulting from the activation of other Ras proteins (i.e. KRAS and NRAS) commonly activated in human cancers remains to be explored.

## Materials and Methods

### Ethics Statement

This study was carried out in accordance with recommendations in the Guide for the Care and Use of Laboratory Animals of the Cincinnati Children’s Hospital Research Foundation. The protocol was approved by the Committee on the Ethics of Animal Experiments of the Cincinnati Children’s Hospital Research Foundation (permit Number:8D06052). When necessary, CO_2_ euthanasia was used. This method was approved by the Animal Care and Use Committee of the Cincinnati Children’s Hospital Research Foundation and consistent with the recommendations of the Panel on Euthanasia of the American Veterinary medical Association.

### Cell Lines

Primary mouse embryonic fibroblasts were isolated from cdc42^f/f^ embryos at embryonic day 12.5 as described previously (Guo JBC 2006). Immortalized cdc42 ^f/f^ fibroblasts were generated through the transduction of cells with retrovirus expressing dominant negative p53 (LXSN-p53dd) followed by selection in 250 µg/ml G418. The dominant negative p53 construct was kindly provided by Moshe Oren of the Weizmann Institute of Science. Immortalized cdc42^f/f^ fibroblasts were transformed through retroviral transduction of either MIEG3-HRasV12 or pKH3-c-Myc and infected cell populations were obtained following fluorescence activated cell sorting (FACS) for GFP 48 hours after infection. All cell lines were cultured in DMEM containing 10% fetal bovine serum, 100 U/ml penicillin/streptomycin and 2 mM L-glutamine and maintained at 37°C and 5% CO_2_.

### Viral Transduction

Conditional cdc42 deletion was achieved following Ad-GFP-Cre infection with Ad-GFP serving as a control. Adenoviral transduction efficiency was monitored by GFP expression and was determined to be >95%. For retroviral transductions, cells were transduced overnight with 4 ml of retroviral-containing media supplemented with 5 µg/ml of polybrene. Pure populations of transduced cells were obtained following antibiotic selection or FACS 48–72 hrs. post-transduction as indicated.

### PCR

Recombination at the cdc42 locus was monitored by genomic PCR. DNeasy Blood and Tissue Kit (Qiagen, Valencia, CA, USA) was used to extract DNA from infected cells or tumor tissue. Primer sequences: CDC42SA2F: AGA CAA AAC AAC AAG GTC CAG AAA C CDC42LA1r: CTG CCA ACC ATG ACA ACC TAA GTT C.

### Immunoblot Analysis and Ras/Cdc42 Pull-down Assay

For immunoblot analysis, total cell lysates were resolved by SDS-PAGE as previously described [41]. Specific proteins were detected using the following primary antibodies: From Cell Signaling Technology (Boston, MA, USA) - Cdc42, p-FAK(Tyr 397), p-MLC(Ser 19), MLC, p-GSK3β(Ser 9), GSK3β, p-PAK1(Thr 423), PAK1, p-Akt(Ser 473), Akt, p-ERK1/2(Thr 202/Tyr 204), ERK1/2, p-MEK1/2(Ser 217/221), MEK, cleaved caspase 3. From Santa Cruz Biotechnology (Santa Cruz, CA, USA)- p16, c-Myc, HA. From BD Biosciences (Franklin Lakes, NJ, USA)- Cdc42, p53. From Bethyl Laboratories (Montgomery, TX, USA)- p-WASP(Ser 483/484). From Oncogene Research Products (La Jolla, CA,USA)- c-H-Ras. From Fitzgerald (Acton, MA, USA)- Gapdh. From Thermo Fisher Scientific (Fremont, CA, USA)- Cyclin D1.

For HRas and Cdc42 pull-down assays, GST- Cells were lysed through direct addition of Lysis Buffer(20 mM Tris-HCl ph 7.6, 100 mM NaCl, 10 mM MgCl_2_, 1% Triton x-100, 0.2% SDS with protease/phosphatase inhibitors) to the culture dish. 200–400 µg of total cell lysate was incubated with 20 µg of glutathione-agarose immobilized GST-fusion proteins (GST-Raf for HRas, GST-PAK1 for Cdc42) for 45 minutes at 4°C. 10% of protein quantity used for pull-down was retained for total input controls. Following incubation, beads were washed three times with wash buffer and bound proteins were resolved by SDS-PAGE and bound/active proteins were detected by immunoblotting.

#### Immunofluorescence and TUNEL staining

Cells were seeded on glass coverslips and fixed with 3.7% formaldehyde. Fixed cells were stained with Rhodamine-phalloidin (Invitrogen, Eugene, OR, USA) and 4,6-diamidino-2-phenylindole (DAPI) (Invitrogen). TdT-mediated dUTP nick end (TUNEL) labeling was carried out using *In Situ* Cell Death Detection Kit, TMR Red (Roche, Indianapolis, IN, USA). Staining was carried out according to manufacturer’s protocol.

### Cell Growth Assay

2×10^5^ cells were seeded in 10 cm dishes in triplicate. Cells were maintained at subconfluence and at days 3, 6, and 9 cells were harvested and viable cells counted on a hemocytometer by trypan blue exclusion.

#### Cell cycle analysis

Subconfluent cells were pulsed with 10 µg/ml BrdU prior to cell harvest and fixation with 100% EtOH overnight at 4°C. Fixed cells were incubated for 30 min. at 37°C in 2N HCl containing 0.5 mg/ml pepsin. Following incubation, acid was neutralized with 0.1M sodium tetraborate. Cells were washed with phosphate buffered saline (PBS) containing 0.5% BSA and PBS +0.5% BSA +0.5% Tween20 before 1 hour incubation with FITC-conjugated anti-BrdU (BD Pharmingen). Stained cells were washed and resuspended in propidium iodide with RNase A for flow cytometric analysis.

### Soft Agar Colony Formation

20,000 cells were seeded per well in a six well dish. Cells were mixed with 0.3% agarose in growth media and layered on top of 0.6% agarose in growth media. Colonies were counted under a light microscope 2–3 weeks post-plating. For each experiment, cells were seeded in triplicate and three fields per well were quantified.

### Xenograft Models

*In vitro* deletion- HRasV12-transformed cells were infected with either Ad-GFP or Ad-GFP-Cre to obtain *cdc42* deletion. 5×10^5^ infected cells were suspended in 200 µl of a 1∶1 PBS:matrigel mix and injected subcutaneously into the flanks of athymic nude mice. Tumor growth was monitored by caliper measurements. Tumor volume was calculated using the formula: volume = 0.52×length×width^2^. Tumors were excised from sacrificed mice at indicated timepoint, weighed, and a portion of the tumors retained for DNA extraction/genotyping, while the remaining tumor was formalin-fixed and paraffin-embedded for sectioning. N = 6.

#### In vivo deletion

3×10^5^ HRasV12;*Cdc42f*/f which were transduced with pBabe-Cre-ERT or control cells were subcutaneously injected into the flanks of athymic nude mice. Five days post-injection, palpable tumors had formed, at which time 100 µl injections of 10 mg/ml 4-OH Tamoxifen (Sigma-Aldrich) in sunflower seed oil was administered daily, five days a week for 3 weeks. Tumor growth was monitored by measurement with calipers. Following three weeks, mice were sacrificed, tumors excised and weighed prior to harvest of tumor tissue for DNA extraction and genotyping. N = 8.

## Supporting Information

Figure S1 **Cdc42 deletion does not induce cell death.** **(a)** Following Cdc42 deletion, cells were fixed and TUNEL staining was performed. **(b)** Following Cdc42 deletion, both adherent and floating cells were harvested for immunoblotting for cleaved caspase 3. Gapdh serves as a loading control.
(EPS)

Figure S2 **Cdc42 overexpression in HRasV12 cells does not increase proliferation, but enhances anchorage-independent cell growth.** **(a)** HRasV12-transformed cells were retrovirally transduced with wild-type Cdc42 or vector control. Cell lysates were blotted for Cdc42. **(b)** An Equal number of Cdc42 overexpressing and vector control cells were plated on day 0. Cells were counted every 3 days by trypan blue exclusion. Experiment was performed in triplicate. **(c)** Vector control and Cdc42-overexpressing cells were grown in 0.3% agarose. Colonies were counted one week and three weeks after plating. Experiments were performed in triplicate with 9 independent fields counted.
(EPS)

Figure S3 **Cdc42 deficiency is selected against in HRasV12 cells, but not c-Myc transformed or non-transformed cell lines.** Following Cdc42 deletion, an equal number of Cdc42+ and Cdc42− cells were seeded. At confluency, cells were passaged and a small number harvested for PCR to assess the relative abundance of the loxp and recombined cdc42 alleles. Cultures were passaged a total of three times. Band intensity was determined using imageJ software, and the percentage of non-recombined to recombined cdc42 was plotted relative to the initial seeding.
(EPS)

Figure S4 **Cdc42 deficiency alters activation of Akt, but not MEK-ERK signaling.** Non-transformed and HRasV12 transformed cells were harvested for western blot analysis following deletion of Cdc42. Membranes were blotted with the indicated antibodies.
(EPS)
